# UTRN inhibits melanoma growth by suppressing p38 and JNK/c-Jun signaling pathways

**DOI:** 10.1186/s12935-021-01768-4

**Published:** 2021-02-04

**Authors:** Sitong Zhou, Wen Ouyang, Xi Zhang, Lexi Liao, Xiaobing Pi, Ronghua Yang, Baiqiang Mei, Huaiyuan Xu, Shijian Xiang, Jiehua Li

**Affiliations:** 1 Department of Dermatology, The First People’s Hospital of Foshan, 81 Lingnan Avenue North, Foshan, 528000 Guangdong China; 2 The Second Clinical Medical College, Zhujiang Hospital, Southern Medical University, Guangzhou, Guangdong China; 3 Department of Dermatology, Binzhou Medical University Hospital, Binzhou, Shandong China; 4 Department of Dermatology, Cosmetology and Venereology, Shenzhen Hospital, Southern Medical University, Shenzhen, Guangdong China; 5 Department of Burn Surgery and Skin Regeneration, The First People’s Hospital of Foshan, Foshan, Guangdong China; 6 Department of Cardiovascular Disease, The First People’s Hospital of Foshan, Foshan, Guangdong China; 7 Department of Bone and Soft Tissue Surgery, Sun Yat-Sen University Cancer Center, Guangzhou, Guangdong China; 8 Department of Pharmacy, Seventh Affiliated Hospital of Sun Yat-Sen University, Shenzhen, 518107 China

**Keywords:** UTRN, Survival analysis, Melanoma, TCGA, GSEA

## Abstract

**Background:**

*Utrophin *(*UTRN*), as a tumor suppressor gene, is involved in various cancer progression. The function of UTRN in the melanoma process and the related molecular mechanisms are still unclear. Herein, we studied the function of UTRN in melanoma growth and the relevant molecular mechanisms.

**Methods:**

Using the GEO database and UCSC Xena project, we compared the expression of UTRN in non-cancerous and melanoma tissues. Immunohistochemistry (IHC) staining, qRT-PCR and Western Blot (WB) were performed to evaluate UTRN expression in clinical samples. A total of 447 cases with UTRN expression data, patient characteristics and survival data were extracted from TCGA database and analyzed. After stable transduction and single cell cloning, the proliferation ability of A375 human melanoma cells was analyzed by Cell Counting Kit‑8 (CCK) and 5‑ethynyl‑2′‑deoxyuridine (EdU) incorporation assays. GSEA was performed to predict the mechanism by which UTRN regulated melanoma growth. Then WB analysis was used to assess the protein expression levels of pathway signaling in overexpression (EXP) melanoma cells. Epac activator 8-pCPT-2′-*O*-Me-cAMP was then used to evaluate the proliferation ability by activation of p38 and JNK/c-Jun signaling pathways.

**Results:**

Data from GEO and UCSC Xena project indicated that UTRN expression was decreased in melanoma. Experiment on clinical samples further confirmed our finding. TCGA results showed that a reduced expression of UTRN in 447 melanoma samples was associated with advanced clinical characteristics (T stage, Clark level, ulceration), shorter survival time and poorer prognosis. In addition, up-regulated UTRN expression inhibited melanoma cell proliferation when compared to control group. MAPK signaling pathway was presented in both KEGG and BioCarta databases by using GSEA tool. WB results confirmed the down-regulated expression of p38, JNK1 and c-Jun in EXP group when compared to control group. Epac activator 8-pCPT-2′-*O*-Me-cAMP treatment could partially rescue proliferation of tumor cells.

**Conclusion:**

We have demonstrated that reduced UTRN predicted poorer prognosis and UTRN inhibited melanoma growth via p38 and JNK1/c-Jun pathways. Therefore, UTRN could serve as a tumor suppressor and novel prognostic biomarker for melanoma patients.

## Background

Cutaneous malignant melanoma, which is responsible for approximately 80% of skin cancer-related deaths, is one of the most aggressive forms of skin cancer. Depending on the clinical stage at diagnosis, 5-year survival rate ranges from 15 to 60% in patients with distant and local metastases [1–3]. Survival rate was associated with tumor characteristics such as Clark level, ulceration and lymph node involvement [4–6]. However, an effective biomarker which can further improve the accuracy of the prognostic indicators mentioned above is still needed. The aim of this study was to contribute to the search for a prognostic molecular marker in melanoma.

*UTRN*, which is located on the chromosomal band 6q24, encodes a component of cytoskeleton utrophin [7]. UTRN plays a role in the organization of connection between cytoskeleton and transmembrane proteins, and can compensate for dystrophin-deficient muscular dystrophy [8]. The expression level of UTRN depends on cell type and tumor. UTRN was found to be significantly decreased and associated with progression in lung cancer and colon cancer, which indicated that UTRN could be a potential tumor suppressor gene [7]. Recent studies also identified that UTRN was decreased in melanoma [7, 9, 10]. However, the link between UTRN and clinical outcome as well as the molecular functional role of UTRN in melanoma have not been made clear.

This work revealed the clinical significance of UTRN expression in melanoma and explored the underlying molecular mechanism. We first clarified that UTRN expression was decreased in melanoma by examining public database and clinical specimens. Then, we analyzed the correlation between UTRN and clinical characteristics as well as survival time in The Cancer Genome Atlas (TCGA). Finally, we found out that UTRN inhibited melanoma growth via p38 and JNK/c-Jun pathways and could act as a tumor suppressing gene.

## Methods

### Patients tissue specimens

Matched pairs of tumor and para-cancer tissues were collected from 8 melanoma patients of the Han Chinese group from 2017 to 2019 (Additional file 1: Table S1). All the patients were proved to have melanoma by histological examination.

### Cell culture

A375 human melanoma cell line was obtained from American Type Culture Collection (ATCC). A375 was maintained in the DMEM (Gibco) supplemented with 10% FBS and cultured at 37 °C in a 5% CO_2_ incubator.

### Quantitative real-time polymerase chain reaction (qRT-PCR)

RNA from fresh samples was extracted using the TRIzol reagent according to the manufacturer’s instructions (Life Technologies). The isolated RNA was used for qRT-PCR experiment using HiScript®II Reverse Transcriptase (VazymE). The qRT-PCR was run on 7500 Real Time PCR System (Applied Biosystems, Foster City, CA) with ChamQ SYBR qPCR Master Mix (VazymE). The primers were listed as follows: UTRN (forward: 5′-CAAACACCCTCGACTTGGTT-3′ and reverse: 5′-TGGTGGAGCTGCTATCAGTG-3′). GAPDH (forward: 5′-TTGATTTTGGAGGGATCTCG-3′ and reverse: 5′-GAGTCAACGGATTTGGTCGT-3′).

### Western Blot (WB)

Protein was extracted using RIPA lysis buffer (Beyotime). Protein concentrations were determined using BCA Protein Assay Kit (Beyotime). Protein samples (25 µg) were loaded and ran on a 10% resolving with 5% stacking SDS PAGE for 1 h at 100 V followed by immersion transfer to a polyvinylidene difluoride membranes (Millipore) at 100 V for 1 h. The membrane was then blocked with 5% skim milk powder in 1xTBST solution for 1 h at room temperature. Primary antibodies against UTRN (1:200, ab95443, Abcam), JNK1 (1:800, 3708, Cell Signaling Technology), p38 (1:200, 27,986, Abcam), c-Jun (1:800, 9165, Cell Signaling Technology) were incubated overnight at 4 °C. The appropriate HRP-conjugated secondary antibodies (mouse anti-rabbit, 1:5000, Abcam; goat anti-mouse, 1:5000, Abcam) were used and incubated at room temperature for 1 h followed by ECL luminol reagent (Tanon) incubation.

### Immunohistochemistry (IHC) staining

A human tissue microarray (TMA, Cat.# ME1004g, Alenabio) was constructed with formalin-fixed paraffin-embedded melanoma tissues and nevus tissues. IHC staining was performed on the TMA by using the appropriate dilatation of primary antibodies (UTRN, 1:600, ab95443, Abcam) followed by incubation with a secondary antibody conjugated with HRP (mouse anti-rabbit, 1:5000, Abcam). TMA was then washed three times with PBS for incubation with AEC (ZSGB-BIO).

### Histologic scoring and analysis

TMA was examined by the cross-product (H score) of the percentage of tumor cell staining at each of the 3 staining intensities. Intensity of immunopositivity was scored as follows: none, 0; weak, 1; moderate, 2; and strong, 3. For example, a particular tumor may have 50% cell staining at intensity = 1 and 50% of cell staining at intensity = 3, for a combined H score of 200 [(50 × 1) + (50 × 3) = 200], which yielded a range from 0 to 300. The final score was graded by H score as follows: Low, H score 0–100; Moderate, H score 101–200; and High, H score 201–300.

### Stable transduction with lentiviral vectors and single cell cloning

The procedures of transduction with lentiviruses and single cell cloning have been described in our previous work [11]. Lentiviruses expressing overexpression (EXP) of UTRN were purchased from Cyagen Bioscience.

### Cell proliferation assay

The cell proliferation was evaluated using Cell Counting Kit-8 (CCK-8, Dojindo) according to the manufacturer’s specification. Briefly, 5 × 10^3^ A375 cells per well were seeded in 96-well plate. To test the growth rate at 24 h, 48 h and 72 h, the number of viable cells in quadruplicate wells was determined using the CCK-8.

### 5‑Ethynyl‑2′‑deoxyuridine (EdU)‑incorporation assays

An EdU labeling kit (cat. no. C 10310‑3; Guangzhou RiboBio Co., Ltd.) was used to investigate cell viability and proliferation in UTRN overexpression melanoma cells. 50 μM EdU labeling media was added in 96‑well plate with stable transduction A375 cells. After fixed with 4% paraformaldehyde, the cells were immunostained with Apollo working solution. Then, the cell nuclei were stained with DAPI solution according to the manufacturer's instructions (cat. no. DA 0001; Beijing Leagene Biotechnology Co., Ltd.). The EdU‑positive cells and DAPI cells in each field were counted and the proliferation rate was calculated from the formula: Proliferation rate = the green fluorescence‑positive cells (proliferating cells)/the blue fluorescence cells (total cells) × 100%.

### Microarray data and RNA-sequencing patient data

The gene expression profiles of melanoma were obtained from the Gene Expression Omnibus (GEO, available at http://www.ncbi.nlm.nih.gov/geo/) database. A previous microarray (GSE3189) data which compared the expression of UTRN between melanoma (n = 45) and nevus (n = 18) patients was downloaded from GEO database. Data was excluded with less than 10 samples. TCGA and GTEx RNA-Seq data were processed by the Toil pipeline and downloaded from the UCSC Xena project (https://xenabrowser.net/datapages/). Wilcoxon rank sum test was performed to compare the expression of UTRN in normal samples obtained from GTEx and TCGA (n = 812) and melanoma samples from TCGA (n = 470).

Clinical information and the RNA-Seq data (470 cases) for melanoma were downloaded from TCGA database (https://genomecancer.ucsc.edu). Patients with incomplete RNA-Seq data (2 cases) or cases with overall survival time less than 1 month (21 cases) were excluded from the analysis. Overall, 447 melanoma cases were enrolled in the study. RNA-Seq data were converted to TPM format and UTRN expression level was retained for further analysis.

### Bioinformatics analysis

The correlation between clinical characteristics and UTRN expression in melanoma patients was analyzed by the Wilcoxon signed-rank test. The samples were then divided into a high and a low UTRN group based on the median value of UTRN. Logistic regression analysis was performed to determine the correlation between UTRN expression and clinical characteristics in the TCGA cohorts. Univariate and Multivariate Cox analysis were used to compare the influence of UTRN expression on survival time along with other clinical characteristics (TNM stage, clinical staging, Clark level, age, ulceration, recurrence, cancer status and race). Significant variables (P ≤ 0.05) found in univariate analysis were further tested in multivariate analysis.

### Gene set enrichment analysis (GSEA)

Using the data obtained from TCGA, GSEA was performed to identify gene sets and pathways associated with UTRN. The expression profile data of 447 cases was sorted by UTRN gene expression value. The melanoma samples were divided into a low expression group and a high expression group according to the median value of UTRN expression. Gene set permutations were performed 1000 times for each analysis. The expression level of UTRN was used as a phenotype label. A normalized enrichment score (NES) is calculated for each gene set based on the size of the set. The differences were considered statistically significant if nominal P value ≤ 0.05 and false discovery rate (FDR) ≤ 25%.

### Statistical analysis

Most of the statistical analyses were done by the bioinformatic tools mentioned above. Statistical analysis for experimental assay was performed with GraphPad Prism 7.0. Differences between the two groups were examined using Student’s t-test. Variables with P value ≤ 0.05 were considered statistically significant (*P ≤ 0.05, **P ≤ 0.01, *** ≤ 0.001).

## Results

### Decreased UTRN expression in melanoma

**Fig. 1:**
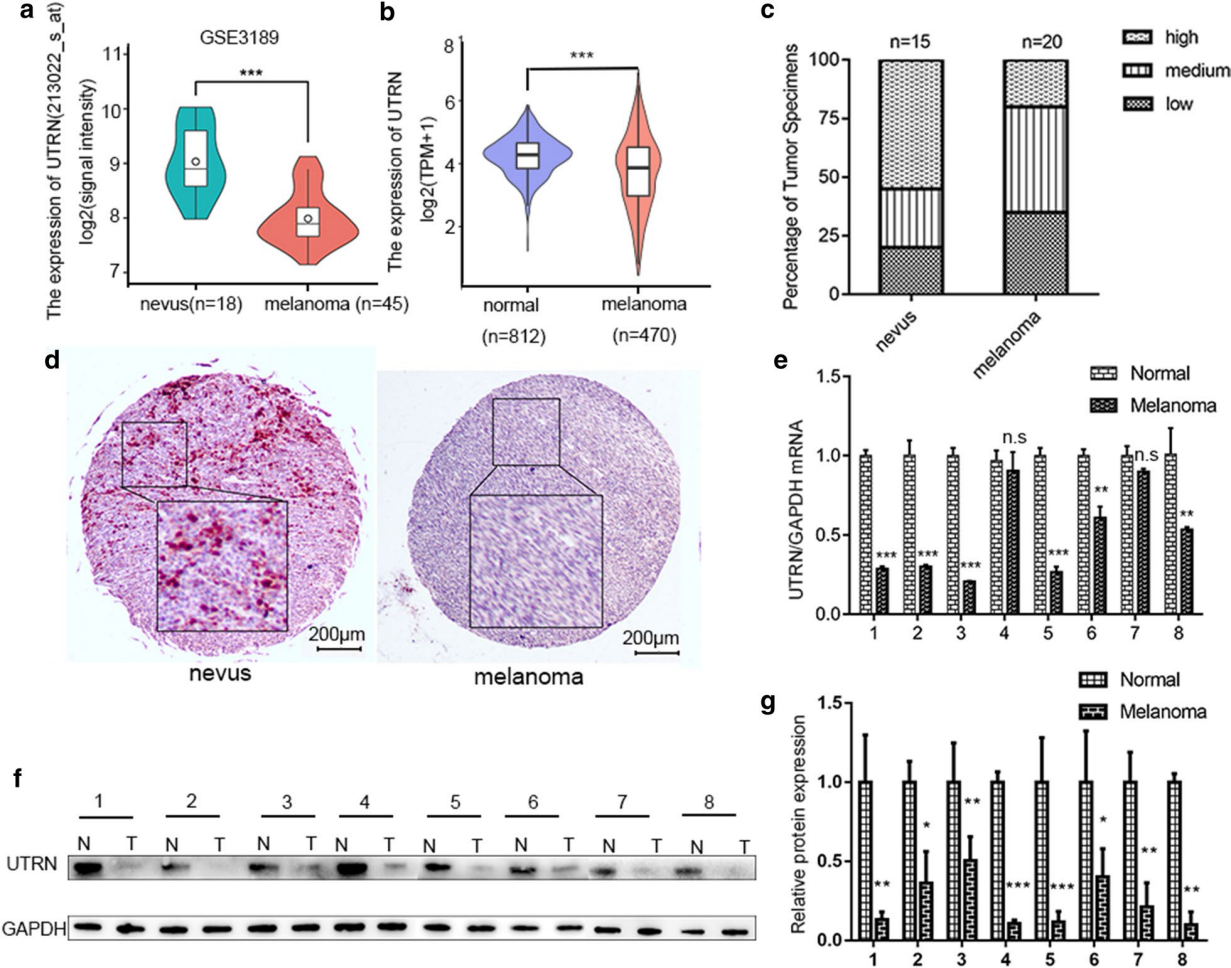
Decreased UTRN expression in melanoma. **a** Analysis of UTRN expression in nevus tissues (n = 18) and melanoma (n = 45) in GEO database (GSE3189) (P < 0.001). **b** Analysis of UTRN expression in normal skin (n = 812) and melanoma (n = 470) from UCSC XENA platform (P < 0.001). **c**, **d** Melanoma tissues (n = 20) presented lower expression of UTRN while benign nevus (n = 15) showed higher expression by IHC staining. **e**–**g** UTRN expression levels measured in 8 paired clinical samples by qRT-PCR (**e**) and Western Blot (**f**, **g**). IHC stain, AEC, scale bar represents 200 μm. Bars represent mean ± SD; *n.s* not significant, *IHC* immunohistochemistry. *P ≤ 0.05, **P ≤ 0.01, ***P ≤ 0.001

To evaluate UTRN expression in melanoma tissues, we first analyzed UTRN mRNA expression from GEO database (GSE3189) (Fig. 1a) and UCSC XENA platform (Fig. 1b). These data showed that UTRN mRNA level was significantly lower in melanoma compared with mRNA level in nevus tissues and normal skin. Next, we evaluated UTRN expression on TMA. The results showed that melanoma tissues presented lower expression of UTRN (n = 20) while benign nevus (n = 15) showed higher expression (Fig. 1c, d). Furthermore, qRT–PCR and WB were conducted to validate UTRN mRNA and protein levels in 8 paired samples of tumor and para-cancerous tissues (Fig. 1e–g, Additional file 5). The results confirmed that UTRN mRNA and protein expression were both decreased in melanoma tissues compared to non-tumorous tissues.

### Patient characteristics

As shown in Table 1, data of 447 melanoma patients with clinical characteristics was downloaded from TCGA. Current prognostic model based on the classic tumor-node-metastasis (TNM) staging is the recognized prognosis standard for melanoma in clinical practice [12]. According to the T-staging classification, 7 patients (0.02%) were in stage Ti (0.02%), followed by 41 (11.48%) in T1, 76 (21.29%) in T2, 89 (24.93%) in T3 and 144 (40.34%) in T4. For N-staging result, 222 patients (64.53%) showed stage N0, 73 (21.22%) showed stage N1 and 49 (14.24%) showed stage N2. For M-staging, 402 patients (95.04%) were classified as stage M0 and 21 (4.96%) as M1 stage. 6 patients (1.49%) were found in Stage 0, followed by 76 (18.81%) in stage I, 133 (32.92%) in Stage II, 169 (41.83%) in Stage III in and 20 (4.95%) in Stage IV. Elevated Clark index has been reported to be associated with poorer prognosis [13–15]. Among 209 melanoma patients, 98 of them (31.72%) were associated with Clark level I–III while the rest 211 (68.29%) were with Clark level IV–V. Melanomas with no ulceration were reported in 144 cases (47.37%) and with ulceration were reported in 160 cases (52.63%). The cancer status was classified as tumor-free in 210 cases (49.65%) and tumor in 213 (50.35%). Most of melanoma cases (74.78%, n = 326) were associated with recurrence. Most patients (97.49%, n = 427) were White people and the rest were Asian people (2.51%, n = 11). The number of patients aged under 60 was 237 (53.02%) and the number of patients aged over 60 was 210 (46.98%). Sites of melanoma were located on extremities (n = 191, 49.35%), head and neck (n = 32, 8.27%) and trunk (n = 164, 42.38%).

**Table 1:** Data of 447 melanoma patients from TCGA

Characteristics	N	Percentages	Characteristics	N	Percentages
T			Clark level		
Ti	7	1.75	I/II/III	98	31.72
T1	41	10.28	IV/V	211	68.29
T2	76	19.05	Ulceration		
T3	89	22.31	No	144	47.37
T4	144	36.09	Yes	160	52.63
N			Cancer status		
N0	222	59.04	Tumor free	210	49.64
N1	73	19.41	With tumor	213	50.35
N2	49	13.03	New tumor event		
M			No	110	25.23
M0	402	95.04	Yes	326	74.77
M1	21	4.96	Race		
Stage			Asian	11	2.51
Stage0	6	1.49	White	427	97.49
Stage1	76	18.81	Age		
Stage2	133	32.92	< 60	237	53.02
Stage3	169	41.83	≥ 60	210	46.98
Stage4	20	4.95	Site		
–	–	–	Extremities	191	49.35
–	–	–	Head and neck	32	8.27
–	–	–	Trunk	164	42.38

Patient characteristics included TNM stage, clinical stage, Clark level, ulceration, cancer status, new tumor event, race, age and site were identified

### Association between UTRN expression and patient clinical characteristics

**Fig. 2:**
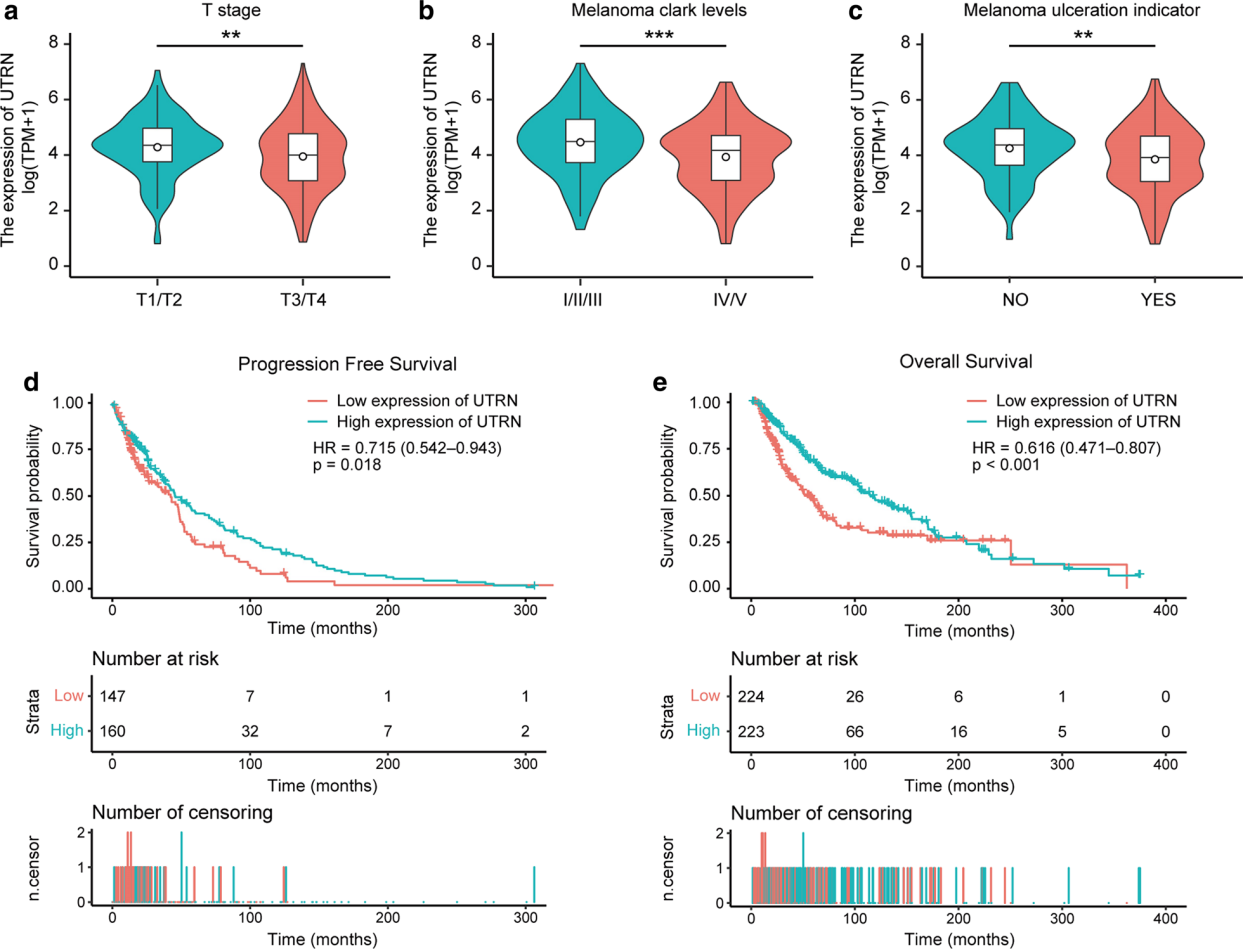
Association with UTRN expression and patient clinical characteristics. The expression of UTRN was correlated to T stage (**a**), Clark level (**b**) and Ulceration (**c**) in melanoma patients in TCGA cohort. **d**, **e** Progression-free survival (**d**) and overall survival (**e**) curves for the patient groups with low and high UTRN mRNA levels were estimated by Kaplan–Meier survival analysis. *TCGA* The Cancer Genome Atlas. *P ≤ 0.05, **P ≤ 0.01, ***P ≤ 0.001

A total of 447 samples from TCGA with UTRN expression and clinical characteristics were analyzed. As shown in Fig. 2a–c, UTRN expression level was associated with clinical characteristics including T stage (T1T2 vs. T3T4, P < 0.01), Clark level (I/II/III vs. IV/V, P < 0.001) and ulceration (No vs. Yes, P < 0.01) in melanoma patients. As shown in Table 2, univariate analysis revealed that expression of UTRN was significantly correlated with T stage [T1T2 vs. T3T4, OR 0.57 (0.36–0.87), P = 0.01], Clark level [I/II/III vs. IV/V, OR 0.54 (0.33–0.88), P = 0.01] and ulceration [No vs. Yes, OR 0.59 (0.37–0.92), P = 0.02]. Notably, ulcerated melanomas have been proved to be significantly associated with more metastasis and shorter survival time [16, 17]. However, no significant differences were found in N stage (N0 vs. N1N2), M stage (M0 vs. M1), site (extremities vs. trunk), age (< 60 vs. ≥ 60), gender (female vs. male), height (< 170 vs. ≥ 170), cancer status (tumor free vs. with tumor), race (Asian vs. White), radiation therapy (No vs. Yes) and weight (< 80 vs. ≥ 80). These results suggested that UTRN expression was related to melanoma progression.

**Table 2:** Logistic regression analysis for UTRN expression and clinical characteristics in TCGA patients

Characteristic	N	OR	P value
T (T1T2 vs. T3T4)	399	0.57 (0.36–0.87)	0.01
N (N0 vs. N1N2)	344	1.05 (0.68–1.64)	0.82
M (M0 vs. M1)	423	1.11 (0.46–2.72)	0.81
Clark level (I/II/III vs. IV/V)	309	0.54 (0.33–0.88)	0.01
Ulceration (no vs. yes)	304	0.59 (0.37–0.92)	0.02
Site (extremities vs. trunk)	355	1.11 (0.73–1.68)	0.64
Age (< 60 vs. ≥ 60)	447	0.78 (0.54–1.14)	0.20
Gender (female vs. male)	447	1.2 (0.82–1.76)	0.35
Height (< 170 vs. ≥ 170)	234	1.52 (0.90–2.55)	0.12
Cancer status (tumor free vs. with tumor)	423	1.34 (0.92–1.97)	0.13
Race (Asian vs. White)	438	1.77 (0.53–6.86)	0.37
Radiation therapy (no vs. yes)	405	1.19 (0.72–1.98)	0.50
Weight (< 80 vs. ≥ 80)	240	178 (1.07–2.98)	0.03

The univariate analysis investigated that UTRN expression was significantly correlated with T stage, Clark level and ulceration in melanoma patients. Categorical dependent variable, greater or less than the median expression level

### Survival outcomes and multivariate analysis

As shown in Fig. 2d, e, lower UTRN expression was found to be associated with poorer progression-free survival (PFS) (P = 0.018) and overall survival (OS) (P < 0.001) when compared with those with higher UTRN expression by Kaplan–Meier survival analysis.

The univariate analysis (Table 3) also confirmed that patients who displayed lower UTRN expression level had poorer outcomes [OS: HR (95% CI) = 0.62 (0.47–0.81), P < 0.001]. Other clinical variables including T stage, N stage, clinical stage, Clark level, age, ulceration, cancer status and race were found to have significant impact on overall survival. In multivariate analysis, UTRN [OS: HR (95% CI) = 0.51 (0.34–0.78), P = 0.002], age [OS: HR (95% CI) = 1.77 (1.12–2.81), P = 0.02], ulceration [OS: HR (95% CI) = 1.67 (1.05–2.66), P = 0.03] and cancer status [OS: HR (95%CI) = 5.82 (3.32–10.21), P < 0.001] were independent prognostic factors in patients with melanoma. Taken together, these results indicated that UTRN level could serve as an independent prognostic factor for melanoma patients.

**Table 3:** Cox proportional-hazard regression analysis for clinical characteristics and overall survival in TCGA patients

Variables	Univariate analysis			Multivariate analysis		
	HR	95% CI	P value	HR	95% CI	P value
UTRN (low vs. high)	0.62	0.47–0.81	< 0.001	0.51	0.34–0.78	0.002
T (T1T2 vs. T3T4)	2.00	1.44–2.77	< 0.001	0.78	0.46–1.34	0.37
N (N0 vs. N1N2)	1.59	1.15–2.19	0.01	1.73	0.52–5.76	0.38
M (M0 vs. M1)	1.74	0.92–3.30	0.09	–	–	–
Stage (stage1/2 vs. stage3/4)	1.64	1.22–2.20	< 0.001	1.06	0.32–3.51	0.93
Clark level (I/II/III vs. IV/V)	2.09	1.47–2.98	< 0.001	1.26	0.75–2.12	0.38
Age (< 60 vs. ≥ 60)	1.59	1.20–2.09	0.001	1.77	1.12–2.81	0.02
Ulceration (no vs. yes)	2.08	1.49–2.91	< 0.001	1.67	1.05–2.66	0.03
New tumor event (no vs. yes)	0.75	0.51–1.09	0.13	–	–	–
Cancer status (tumor free vs. with tumor)	4.27	3.00–6.07	< 0.001	5.82	3.32–10.21	< 0.001
Race (Asian vs. White)	0.22	0.10–0.48	< 0.001	0.55	0.07–4.17	0.56

The univariate analysis presented impact of UTRN expression, T stage, N stage, M stage, clinical stage, Clark level, age, ulceration, new tumor event, cancer status and race on overall survival in melanoma. Additionally, the multivariate analysis demonstrated the significance of the effect of UTRN expression, age, ulceration and cancer status on overall survival in melanoma

### UTRN inhibited proliferation of melanoma cells

**Fig. 3:**
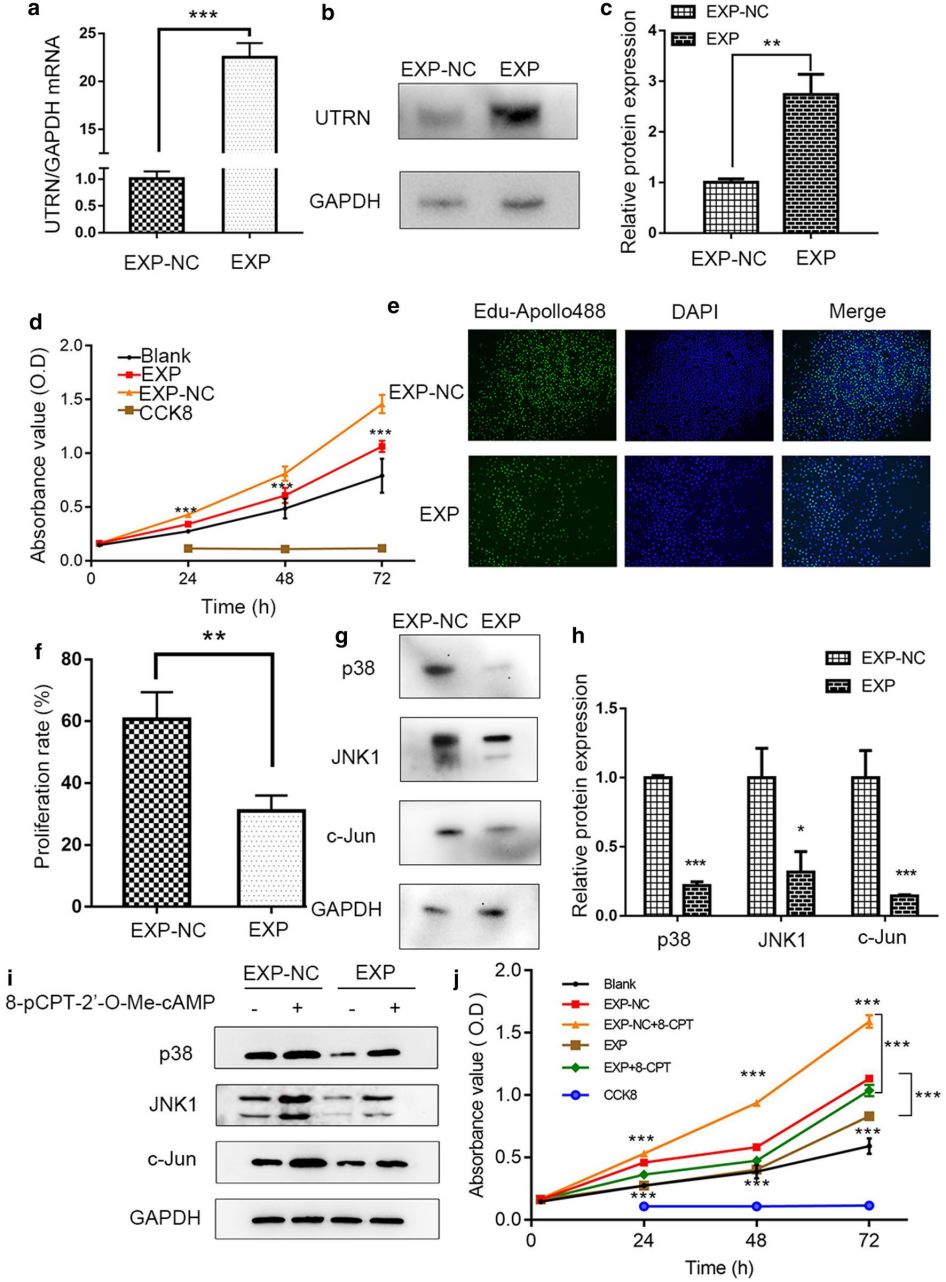
UTRN inhibited proliferation via p38 and JNK1/c-Jun pathways in A375 melanoma cell. **a**–**c** qRT-PCR (**a**) and WB (**b**, **c**) results showed UTRN expression after stable transduction with lentiviral vectors. **d** CCK-8 assay of A375 cell line including EXP, EXP-NC and Blank control groups. **e**, **f** EdU incorporation assay in A375‑EXP‑UTRN transfected cells and negative controls. Magnification, × 200. **g**, **h** WB results showed that p38, JNK and c-Jun were down-regulated in EXP group. **i** After treatment with 8-pCPT-2′-*O*-Me-cAMP (100 μM, 30 min), the levels of p38, JNK and c-Jun were enhanced. **j** The proliferative ability was increased after 8-pCPT-2′-*O*-Me-cAMP treatment. 8-CPT: 8-pCPT-2′-*O*-Me-cAMP. Data was analyzed by the Student’s t-test. Bars represent mean ± SD; *P ≤ 0.05, **P ≤ 0.01, ***P ≤ 0.001

After stable transduction and single cell cloning, qRT-PCR and WB tests showed that mRNA and protein expression of UTRN were prominently altered in EXP group compared to control group (Fig. 3a–c). These successfully constructed recombinant lentiviral vectors lay down a strong foundation for further investigation into the possible role of UTRN in melanoma development.

We next examined the effects of UTRN on cell proliferation. CCK-8 and EdU incorporation assays were used to study the effect of UTRN expression on melanoma cell proliferation. As shown in Fig. 3d–f, cell viability was significantly decreased in EXP group compared to control group.

### UTRN down-regulated p38 and JNK/c-Jun molecular signaling in melanoma

To investigate the function of UTRN in melanoma, the effects of UTRN expression on various biological functional gene sets were analyzed by the GSEA approach. In the GSEA analysis of KEGG, BioCarta and Reactome enrichment (Additional file 2: Figure S1, Additional file 3: Figure S2, Additional file 4: Table S2), higher UTRN expression was involved in TGF-beta signaling pathway in KEGG enrichment analysis, Wnt signaling pathway in KEGG enrichment analysis, ErbB signaling pathway in KEGG enrichment analysis, MAPK signaling pathway in KEGG and BioCarta enrichment analysis, apoptosis pathway in KEGG enrichment analysis, Fas pathway in BioCarta enrichment analysis, PTEN pathway in BioCarta enrichment analysis, pathways in cancer in KEGG enrichment analysis, Toll-like receptor signaling pathway in KEGG, BioCarta and Reactome enrichment analysis, T cell receptor signaling pathway in KEGG enrichment analysis, B cell survival pathway in BioCarta enrichment analysis, ILS signaling pathway in Reactome enrichment analysis and IL2RB pathway in BioCarta enrichment analysis. Notably, it could be seen that MAPK signaling pathway was presented in both KEGG and BioCarta databases.

We subsequently identified hub genes in MAPK pathways and confirmed their differential expression. We focused on MAPK pathway which includes the signaling molecules p38, JNK and c-Jun. WB assay was performed and the results confirmed down-regulated expression of p38, JNK1 and c-Jun in UTRN-EXP group when compared to control group (Fig. 3g, h). Then, cells were treated for 30 min with the Epac-selective agonist (8-pCPT-2′-*O*-Me-cAMP, 100 μM) as a p38, JNK/c-Jun activator to rescue p38, JNK1 and c-Jun expressions [11]. The results indicated that upon addition of activator, p38, JNK1 and c-Jun were upregulated (Fig. 3i). To further verify whether UTRN mediated melanoma proliferative phenotype through p38, JNK/c-Jun pathways, we investigated cell proliferation activity when exposed to 8-pCPT-2′-*O*-Me-cAMP. This treatment could partially rescue cell proliferation by altering p38, JNK/c-Jun activity (Fig. 3j). These data confirmed our hypothesis that p38, JNK/c-Jun activity was responsible for UTRN-mediated growth in melanoma.

## Discussion

UTRN, an autosomal homologue of dystrophin, is expressed in several tissues especially in mature skeletal muscle [18]. Recent studies have shown that UTRN plays an important role in various important cellular processes, such as the development of neuromuscular junctions, cell signaling, adhesion and apoptosis [7, 18, 19]. However, the link between UTRN and clinical characteristics as well as its biological function in melanoma has remained unclear.

Our study further confirmed that UTRN expression was decreased in melanoma. Additionally, bioinformatic analysis demonstrated that decreased expression of UTRN in melanoma was associated with advanced clinical characteristic (T stage, Clark level, ulceration), lower survival time and poorer prognosis. Functionally, up-regulated UTRN inhibited proliferation in A375 melanoma cell. To identify the underlying mechanism of UTRN in melanoma, GSEA using TCGA data and experimental assays were performed to elucidate that UTRN might regulate melanoma growth through p38 and JNK/c-Jun pathways.

Recent studies have demonstrated that UTRN levels were decreased in various cancers including lung cancer, colon cancer, stomach cancer, etc. [7]. Interestingly, some studies showed that UTRN expression was higher in the plasma of breast cancer patients and ovarian cancer patients, and UTRN membranous localization was significantly increased in pancreatic endocrine tumor [20, 21]. Moreover, another research showed that UTRN expression did not change in peripheral T-cell lymphomas compared to that in the normal T-cells [22]. These results indicated that UTRN expression depended on the type of cancer and cells. Some studies also illustrated UTRN expression level in melanoma. Li et al. [7] reported that inactivation mutations of UTRN were found in melanoma cases (4/15). Another study showed that UTRN was a down-regulated gene by using microarray data from four melanoma-derived cell line pairs [9]. Similar results were reported in a study using microarray-based CGH [10]. Consistent with these results, we confirmed that melanoma tissues presented lower UTRN expression levels than non-tumorous tissues.

Ulceration in melanoma has been well-recognized as a prognostic factor for melanoma candidate selection [16, 23]. Ulcerated melanoma is associated with a higher risk of metastasis, a lower OS and PFS in patients. The most recent version of the Staging System proposed by the American Joint Committee on Cancer (AJCC8) has concluded that ulceration is an independent adverse prognostic factor in melanoma [24]. In our study, UTRN expression was significantly correlated with ulceration. Previous studies reported that race and age could also be important factors which affected melanoma patients' prognosis [25]. Asian people are more likely to have a lower 5-year survival rate than White people, which is most likely due to delay diagnosis. Elderly people tend to have a more aggressive primary tumor, which present thicker depth and higher mitotic rates. Consistent with these results, our data confirmed that Asian people and elderly people had a poorer outcome when compared to White people and younger people, respectively.

The loss of heterozygosity (LOH) is a common occurrence in human tumors, which indicates the absence of a functional tumor suppressor gene in the lost region. Higher LOH rate was found at UTRN, which was proved to be significantly associated with poor prognostic factors including high TNM stages, high T stages, positive lymph node status, an unfavorable disease course, and the presence of > 10% solid growth pattern [26]. This finding strongly suggested that UTRN could function as a prognostically important tumor suppressor gene. Consistent with this function, a study showed that overexpression of UTRN resulted in inhibition of tumor cell growth [7]. However, another study showed that membranous UTRN was related to higher Ki-67 proliferative indices in pancreatic endocrine tumors [21]. In our study, up-regulated UTRN expression inhibited the proliferation of human melanoma cells. Taken together, maintaining UTRN activity was important for cell function.

To further investigate the functional mechanism of UTRN in melanoma, GSEA using TCGA data revealed that UTRN might be associated with MAPK signaling pathway. The findings of immunoblot assays demonstrated that up-regulated UTRN inactivated p38 and JNK/c-Jun pathways, which were the two MAPK classical signal transduction pathways. 8-pCPT-2′-*O*-Me-cAMP, a specific activate regulator of Epac, has been proved to be upregulated p38 and JNK/c-Jun expressions successfully [11]. After exposed to 8-pCPT-2′-*O*-Me-cAMP, our results confirmed that p38, JNK/c-Jun activity was responsible for UTRN-mediated growth in melanoma. Recent research revealed that MAPK signaling pathway played crucial roles in the pathogenesis of melanoma and most melanomas exhibited constitutive activation of the MAPK pathway [27]. JNK activation was associated with cell proliferation and a shorter relapse-free period in malignant melanoma [28]. Conversely, inhibition of JNK/c-Jun signaling induced cell apoptosis [29]. In addition, activated p38 contributed to cell migration and in vivo growth of melanoma [30], while inhibition of p38 reduced melanoma cell proliferation [31]. Intriguingly, one study showed that SAPK/p38-MAPK signaling pathways were constantly activated in the hearts of utrophin–dystrophin knockout mice [32]. Similarly, our study also showed that upregulation of UTRN induced a reduction in the protein expression of p38, which indicated that p38 could act as a candidate protein for UTRN-modulated biological process.

This study had several limitations. The clinical information from the TCGA database was not comprehensive. Some information of melanoma patients, such as treatment information, tumor size and histological differentiation, was not available on the TCGA website. In future study, more clinical cases with follow-up and complete information will be collected to conduct a more comprehensive analysis of UTRN in melanoma.

## Conclusions

In conclusion, our study revealed that UTRN was decreased in melanoma, and lower expression of UTRN was related to advanced clinical characteristic and unfavorable prognosis. Moreover, experimental assays identified that UTRN inhibited melanoma proliferation ability. In addition, p38 and JNK/c-Jun signaling pathways were the key biological mechanisms regulated by UTRN in melanoma. Collectively, this work discovered that UTRN could act as a tumor suppressor gene in melanoma and serve as a prognostic factor.

## Supplementary Information

**Additional file 1: Table S1.** Clinical characteristics of melanoma patients.

**Additional file 2: Figure S1.** GSEA-KEGG pathway analysis of UTRN expression in melanoma patients. (**a–h**) In the GSEA analysis of KEGG enrichment, TGF-beta pathway (**a**), Wnt pathway (**b**), ErbB pathway (**c**), MAPK pathway (**d**), apoptosis process (**e**), pathways in cancer (**f**), Toll pathway (**g**) and T cell receptor signaling pathway (**h**) are differentially enriched in UTRN-related melanoma patients. ES, enrichment score; NES, normalized ES; FDR, false discovery rate.

**Additional file 3: Figure S2.** GSEA-BioCarta and Reactome pathway analyses of UTRN expression in melanoma patients. (**a–h**) GSEA results Toll pathway in BioCarta (**a**) and Reactome enrichment analysis (**b**), MAPK pathway in BioCarta enrichment analysis (**c**), Fas pathway in BioCarta enrichment analysis (**d**), PTEN pathway in BioCarta enrichment analysis (**e**), ILS pathway in Reactome enrichment analysis (**f**), IL2RB pathway in BioCarta enrichment analysis (**g**) and B cell survival pathway in BioCarta enrichment analysis (**h**) are differentially enriched in UTRN-related melanoma patients. ES, enrichment score; NES, normalized ES; FDR, false discovery rate.

**Additional file 4: Table S2.** Gene sets enriched in phenotype high.

**Additional file 5.** The amplification file for the qPCR analysis.

## Data Availability

All mRNA-seq data and clinical information (447 cases) are available in TCGA. The gene expression profiles of melanoma were obtained from the GEO (GSE3189), available at http://www.ncbi.nlm.nih.gov/geo/. The melanoma and normal skin RNA-sequence data based on TCGA and GTEx was downloaded from the UCSC Xena database (https://xenabrowser.net/datapages/).

## Abbreviations

UTRN: Utrophin
TCGA: The Cancer Genome Atlas
GSEA: Gene set enrichment analysis
qRT-PCR: Quantitative real-time polymerase chain reaction
WB: Western Blot
GEO: Gene Expression Omnibus
PFS: Progression-free survival
OS: Overall survival
NES: Normalized enrichment score
TNM: Tumor-node-metastasis
LOH: Loss of heterozygosity
TLR: Toll-like receptor
TMA: Tissue microarray
IHC: Immunohistochemistry
EdU: 5‑Ethynyl‑2′‑deoxyuridine
EXP: Overexpression
AJCC: American Joint Committee on Cancer
