# Supplementary material for: UTRN inhibits melanoma growth by suppressing p38 and JNK/c-Jun signaling pathways
Source: Cancer Cell Int. 2021 Feb 4;21:88. doi: 10.1186/s12935-021-01768-4 (PMC7905598; doi:10.1186/s12935-021-01768-4)
Supplement: Supplementary file 1 — Additional file 1: Table S1. Clinical characteristics of melanoma patients. [file 12935_2021_1768_MOESM1_ESM.docx]

| **ID** | **Sex** | **Age, y** | **Location** | **Primary/Metastasis** |
| --- | --- | --- | --- | --- |
| Case 1 | M | 64 | Right plantar | Metastasis |
| Case 2 | M | 56 | Right plantar | Metastasis |
| Case 3 | F | 78 | Right plantar | Metastasis |
| Case 4 | F | 68 | Left plantar | Primary |
| Case 5 | F | 75 | Left ankle | Primary |
| Case 6 | M | 49 | Right plantar | Primary |
| Case 7 | F | 68 | Left plantar | Primary |
| Case 8 | F | 50 | Right plantar | Primary |

Table S1: Clinical characteristics of melanoma patients.

The Clinical Characteristic of 8 melanoma patients were identified. Abbreviations: F, female; M, male.
