# Supplementary figures and images for: UTRN inhibits melanoma growth by suppressing p38 and JNK/c-Jun signaling pathways
Source: Cancer Cell Int. 2021 Feb 4;21:88. doi: 10.1186/s12935-021-01768-4 (PMC7905598; doi:10.1186/s12935-021-01768-4)

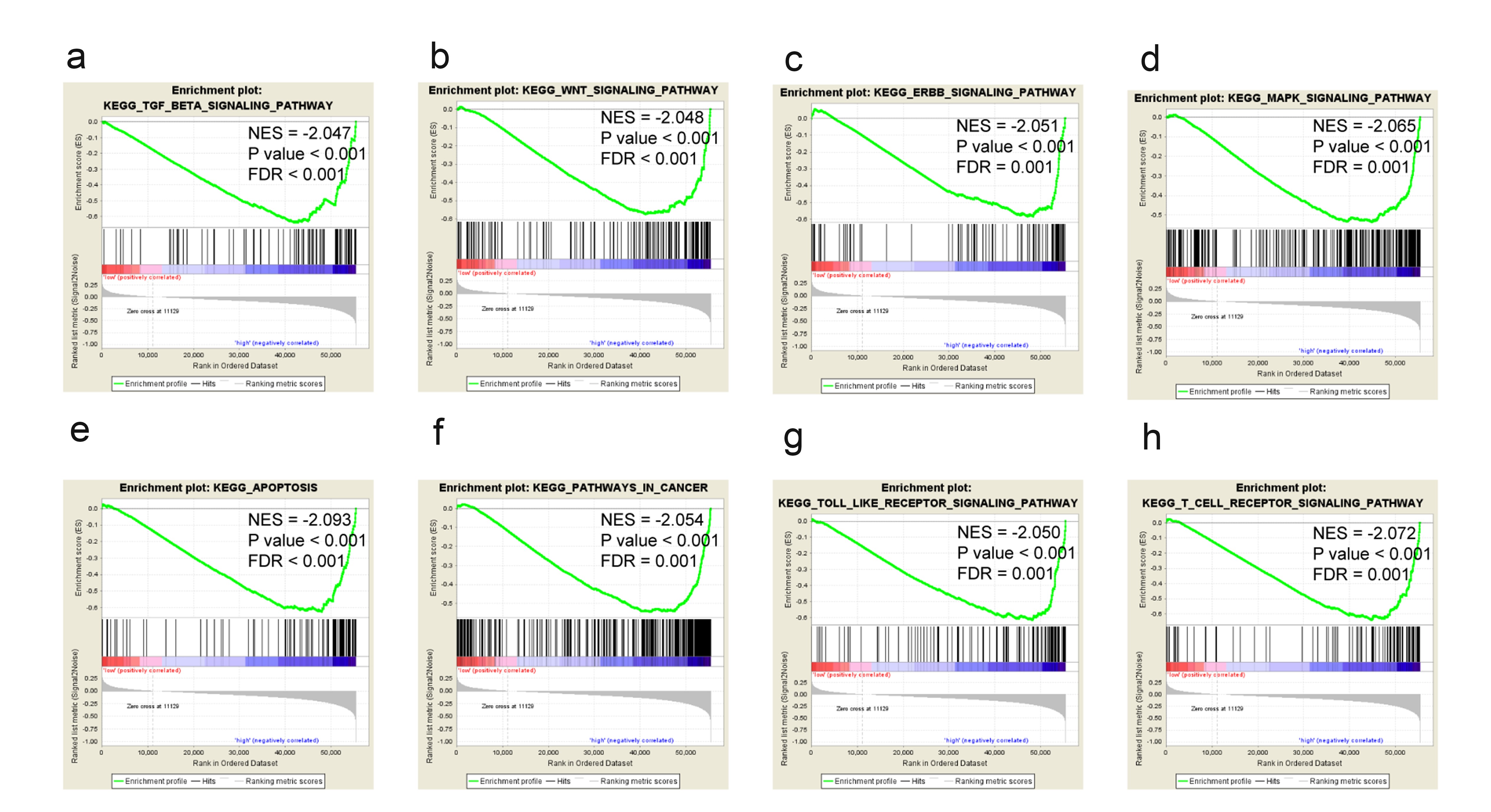

Supplement: Supplementary file 2 — Additional file 2: Figure S1. GSEA-KEGG pathway analysis of UTRN expression in melanoma patients. (a–h) In the GSEA analysis of KEGG enrichment, TGF-beta pathway (a), Wnt pathway (b), ErbB pathway (c), MAPK pathway (d), apoptosis process (e), pathways in cancer (f), Toll pathway (g) and T cell receptor signaling pathway (h) are differentially enriched in UTRN-related melanoma patients. ES, enrichment score; NES, normalized ES; FDR, false discovery rate. [file 12935_2021_1768_MOESM2_ESM.jpg]

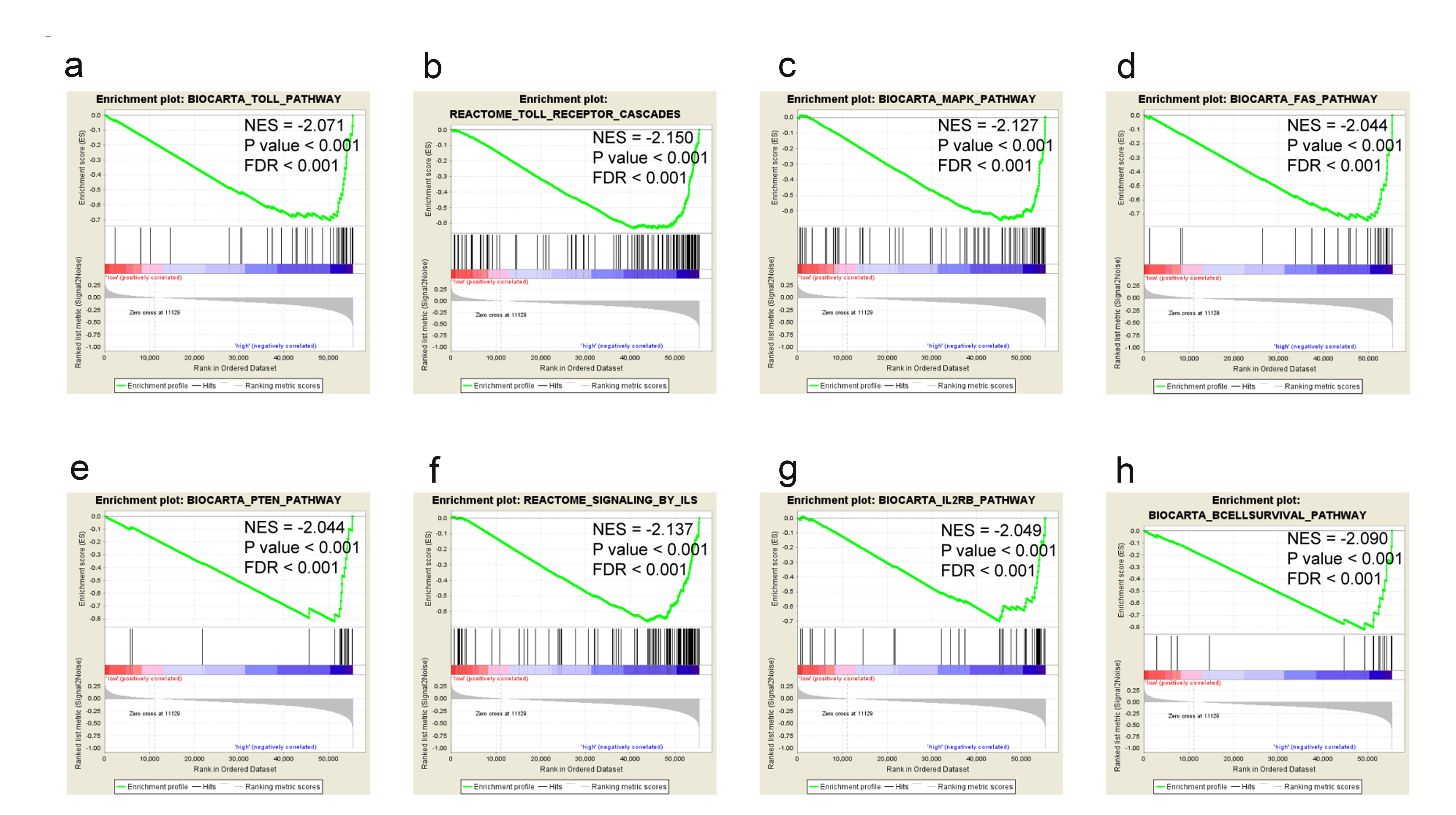

Supplement: Supplementary file 3 — Additional file 3: Figure S2. GSEA-BioCarta and Reactome pathway analyses of UTRN expression in melanoma patients. (a–h) GSEA results Toll pathway in BioCarta (a) and Reactome enrichment analysis (b), MAPK pathway in BioCarta enrichment analysis (c), Fas pathway in BioCarta enrichment analysis (d), PTEN pathway in BioCarta enrichment analysis (e), ILS pathway in Reactome enrichment analysis (f), IL2RB pathway in BioCarta enrichment analysis (g) and B cell survival pathway in BioCarta enrichment analysis (h) are differentially enriched in UTRN-related melanoma patients. ES, enrichment score; NES, normalized ES; FDR, false discovery rate. [file 12935_2021_1768_MOESM3_ESM.jpg]
