# Supplementary material for: UTRN inhibits melanoma growth by suppressing p38 and JNK/c-Jun signaling pathways
Source: Cancer Cell Int. 2021 Feb 4;21:88. doi: 10.1186/s12935-021-01768-4 (PMC7905598; doi:10.1186/s12935-021-01768-4)
Supplement: Supplementary file 4 — Additional file 4: Table S2. Gene sets enriched in phenotype high. [file 12935_2021_1768_MOESM4_ESM.docx]

| **Gene set name** | **Gene set collection** | **NES** | **NOM**  **p-value** | **FDR**  **q-value** |
| --- | --- | --- | --- | --- |
| TGF-beta | KEGG | -2.05 | <0.001 | <0.001 |
| Wnt | KEGG | -2.05 | <0.001 | <0.001 |
| ErbB | KEGG | -2.05 | <0.001 | 0.001 |
| MAPK | KEGG | -2.07 | <0.001 | 0.001 |
|  | BioCarta | -2.13 | <0.001 | <0.001 |
| Apoptosis | KEGG | -2.09 | <0.001 | <0.001 |
| Fas | BioCarta | -2.04 | <0.001 | <0.001 |
| PTEN | BioCarta | -2.04 | <0.001 | <0.001 |
| Pathways in cancer | KEGG | -2.05 | <0.001 | 0.001 |
| TOLL | KEGG | -2.05 | <0.001 | 0.001 |
|  | BioCarta | -2.07 | <0.001 | <0.001 |
|  | Reactome | -2.15 | <0.001 | <0.001 |
| T cell recptor signaling | KEGG | -2.07 | <0.001 | 0.001 |
| B cell survival | BioCarta | -2.09 | <0.001 | <0.001 |
| ILS | Reactome | -2.14 | <0.001 | <0.001 |
| IL2RB | BioCarta | -2.05 | <0.001 | <0.001 |

Table S2: Gene sets enriched in phenotype high.

In the GSEA analysis of KEGG, BioCarta and Reactome enrichment, higher UTRN expression was involved in TGF-beta, Wnt, ErbB, MAPK, apoptosis, Fas, PTEN, pathways in cancer, Toll-like receptor, T cell receptor, B cell survival, ILS and IL2RB pathways. NES: normalized enrichment score; NOM: nominal; FDR: false discovery rate. Gene sets with NOM p-value<0.05 and FDR q-value <0.25 are considered as significant.
